# Surveillance of laboratory exposures to human pathogens and toxins, Canada, 2022

**DOI:** 10.14745/ccdr.v49i09a06

**Published:** 2023-09-01

**Authors:** Christine Abalos, Audrey Gauthier, Antoinette Davis, Cailey Ellis, Nathalie Balbontin, Aryan Kapur, Samuel Bonti-Ankomah

**Affiliations:** 1 Health Security Regional Operations Branch, Public Health Agency of Canada, Ottawa, ON

**Keywords:** Centre for Biosecurity, human pathogens and toxins, laboratory-acquired infections, laboratory exposures, laboratory incidents, Laboratory Incident Notification Canada, surveillance

## Abstract

**Background:**

The Laboratory Incident Notification Canada (LINC) surveillance system was launched in 2015 to monitor the mandated national reporting of laboratory incidents. This report describes the laboratory exposures reported in 2022.

**Methods:**

Exposure incidents were analyzed by activity, occurrence, sector, root cause and pathogens/toxins implicated, while affected individuals were analyzed by education, exposure route, role and years of laboratory experience. An analysis of the median number of exposures per month was conducted, and time between the exposure incident date and the date the incident was reported to LINC was examined.

**Results:**

Forty confirmed laboratory exposure incident reports were received, with two suspected laboratory-acquired infections. The exposure incident rate per 100 active licences was 3.8, and the number of exposure incidents was highest in September. The majority of exposure incidents involved risk group 2 pathogens (n=27; 63%) and non-security sensitive biological agents (n=36; 84%). Microbiology was the most cited activity occurring during the exposure event (n=20; 50%), and sharps and procedure-related issues were the most common occurrences (n=15; 24.2% each). Most incidents were reported by the academic sector (n=16; 40%). Human interaction was the most common root cause (n=20; 23.8%) and most affected individuals were technicians/technologists (n=68; 73.1%). The median time delay between the incident date and reporting date was 5.5 days.

**Conclusion:**

The exposure incident rate was lower in 2022 than in 2021. Incidents related to sharps and standard operating procedures remained the most common occurrence types. The most cited root cause of exposure incidents involved human interaction.

## Introduction

The accidental release or improper disposal of human pathogens and toxins (HPTs) can pose a biosafety or biosecurity threat to the laboratory personnel working with these agents, as well as the Canadian population in general. To improve the safety and security of laboratory personnel working with HPTs and protect the public from the risks posed by exposure to HPTs, the *Human Pathogens and Toxins Act* (HPTA) and the *Human Pathogens and Toxins Regulations* (HPTR) were enacted in Canada in 2015 ((1)).

The HPTA classifies HPTs into four groups based on the level of risk they present to an individual and the community, with risk group 1 (RG1) pathogens being those pathogens that have little to no individual or community risk; risk group 2 (RG2) pathogens posing a moderate individual risk and low community risk; risk group 3 (RG3) pathogens posing a high individual risk and a low community risk; and risk group 4 (RG4) pathogens posing both a high individual and community risk ((2)). Under the HPTA, all laboratories conducting controlled activities with HPTs, such as possessing, producing, storing, transferring or disposing of HPTs, must acquire a licence, unless an exclusion has been granted ((3)), and the reporting of incidents involving RG2, RG3 and RG4 pathogens is mandatory, unless the agent or incident falls outside the scope of the HPTA.

In 2015, the Public Health Agency of Canada (PHAC) established the Laboratory Incident Notification Canada (LINC) surveillance system to oversee the reporting of laboratory incidents involving RG2, RG3 and RG4 HPTs by regulated parties, in accordance with the HPTR. These incident reports not only allow for the identification, monitoring and analysis of trends related to exposures, but also ensure that an appropriate follow-up response and evidence-based recommendation can be provided to facilities by PHAC’s biocontainment inspectors to help minimize health risks and reduce the likelihood of similar incidents in the future. The data from these reports also inform the development of resources and tools by LINC to fill knowledge gaps and raise awareness of biosafety practises in laboratories.

Outside of Canada, there are surveillance systems that exclusively monitor agents that have the potential to pose a high biosecurity risk. In the United States, the Federal Select Agent Program, which was brought about as part of the *Public Health Security and Bioterrorism Preparedness and Response Act of 2002* ((4)), oversees the possession, usage and relocation of select agents and toxins that have the potential to pose a significant threat to the public ((5)). In Australia, the *Security Sensitive Biological Agent Standards* outline the requirements for the safe handling, storage, and removal of known or suspected security sensitive biological agents (SSBAs) within qualified facilities ((6)). Security sensitive biological agents are a subset of RG3 and RG4 human pathogens and prescribed toxins that have been determined to pose an increased biosecurity risk due to their potential for use as a biological weapon ((2)). Handling of SSBAs in Australia is managed by the Australian Department of Health and Aged Care, while other Australian agencies such as the Department of Agriculture, Fisheries and Forestry, the Department of Defence and the Department of Home Affairs monitor the importation and exportation of these agents ((7)). In contrast to these systems, LINC gathers and reviews data from reports on incidents involving a wide range of HPTs, not exclusively on SSBAs ((8)).

The aim of this report is to share data on laboratory exposure incidents that occurred in 2022 and inform laboratory safety measures by increasing awareness of the risks associated with working with HPTs and highlighting potential areas of concern. Exposure incidents are described by sector, HPT, occurrence type, main activity and root cause. The affected individuals will also be described by their role, level of education and years of experience.

## Methods

### Data sources

The LINC surveillance system monitors exposure, non-exposure, and other incidents in laboratories in Canada regulated under the HPTA and HPTR. Under the HPTA and HPTR, an exposure incident is defined as a laboratory incident that could have resulted in intoxication/infection or did result in a suspected or confirmed laboratory-acquired infection (LAI) ((9)). A non-exposure incident refers to any of the following: 1) the inadvertent possession, production or release of a pathogen or toxin; 2) a missing, lost or stolen pathogen or toxin; or 3) an SSBA not being received within 24 hours of expected arrival.

After a laboratory incident has occurred, the laboratory must complete a standardized form through PHAC’s Biosecurity Portal and include specific information about the incident. Data are then captured using the Microsoft Customer Relationship Management system and reviewed for accuracy by a LINC team member. Data from exposure incidents that occurred between January 1, 2022, and December 31, 2022, as well as incidents with an unknown incident date that were submitted in the Biosecurity Portal in this timeframe, were retrieved and analyzed for this annual report. Data from the most recently submitted follow-up reports were used for analysis if multiple follow-up reports were submitted for a particular incident. In addition, if no follow-up report was submitted, data from the initial incident report were used. After extracting the data, outliers were investigated and duplicate entries were removed. The submission of an incident report involving agents classified as RG1 or in their natural environment are not required under the HPTA/HPTR and are considered as voluntary reports. Such incidents are often incomplete and were not included in the analysis for this report.

### Analysis

Data from the LINC surveillance system were extracted on February 8, 2023, from PHAC’s Biosecurity Portal, validated using Microsoft Excel, and descriptive statistics were computed using R 4.1.1. Exposure incidents, including suspected and confirmed LAIs, were classified as confirmed or ruled out after investigation of the incident in the follow-up reports. If an exposure was ruled out, or if it was confirmed that the person was not exposed to the HPT, the affected persons in that report were also ruled out. Because regulated parties can update and provide details in their previously submitted reports at any time, data from reports received between 2016 and 2021 were reanalyzed. As a result, minor differences may exist between the values found in this year’s annual report and those from previous years.

This annual report is focused on confirmed exposure incidents. Of the confirmed exposure incidents, analysis was done at the level of the active licence holder and at the level of the affected person. The former included the distribution of incidents by sector, main activity, root cause(s), occurrence type and implicated pathogen/toxin. The latter examined distribution by highest level of education, years of experience, route of exposure, sector and main role.

The exposure incident rate per 100 active licences for 2016 to 2022 was also calculated and displayed, overlaying the trend of exposure incidents over time throughout these years. The exposure incident rate was calculated by dividing the number of exposure incidents reported during a one-year period by the total number of active licences in a one-year period and multiplying by 100 active licences ((10)). Finally, the median monthly number of exposure incidents of all previous years of the LINC program was compared to the number of monthly exposures in 2022. A median rather than a mean was calculated since this measure reduces noise from outlier data and offers a better measure of the central tendency of exposure incidents.

## Results

**Figure 1:**
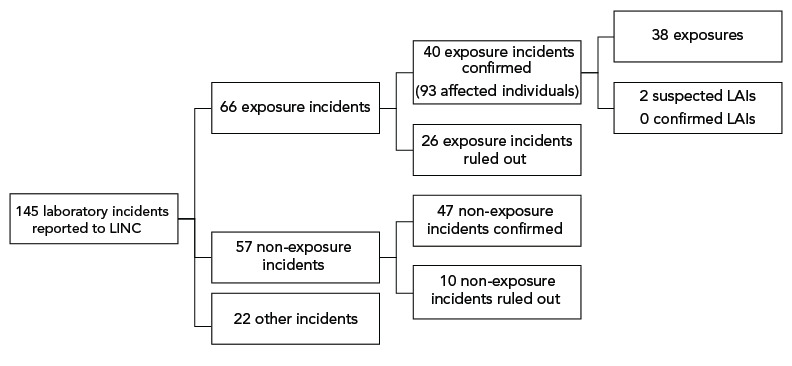
Types of incidents reported to Laboratory Incident Notification Canada and exposure incidents included in analysis, Canada, 2022 Abbreviations: LAIs, laboratory-acquired infections; LINC, Laboratory Incident Notification Canada

There were 145 reports of laboratory incidents received between January 1 and December 31, 2022. Of these reports, 66 were exposure reports, 57 were non-exposure reports and 22 were other reports involving changes in biocontainment (**Figure 1**). Of the 66 reported exposure incidents, 40 were confirmed and 26 were ruled out. Two of the confirmed exposure incidents were suspected LAIs (Figure 1). Out of the 57 non-exposure incident reports received, 47 were confirmed and 10 were ruled out. While 94 people were initially reported as being exposed through these laboratory incidents, one person was later ruled out, bringing the total to 93 exposed people in 2022.

**Figure 2:**
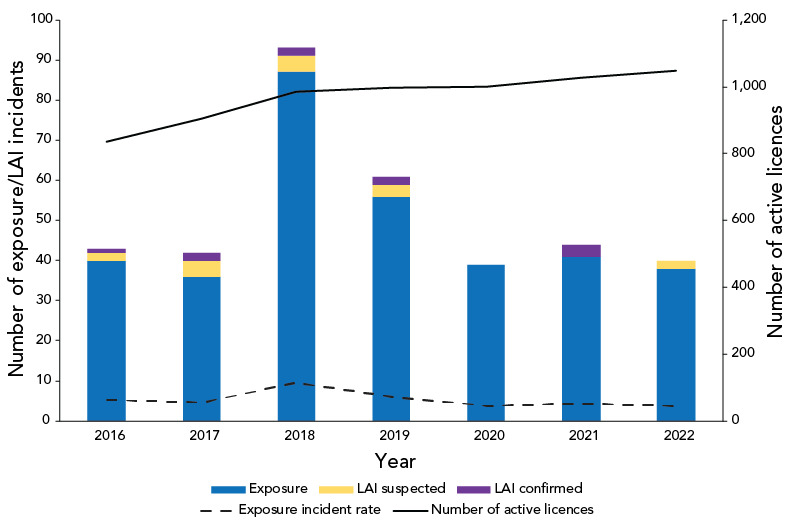
Confirmed exposure incidents, suspected and confirmed laboratory-acquired infections and exposure incident rate, Canada, 2016–2022 Abbreviation: LAI, laboratory-acquired infection

In 2022, there were 1,048 active licences held by laboratories working with HPTs in Canada, which means that for every 100 active licences, the exposure incident rate was 3.8 (**Figure 2**). This is the lowest rate observed since 2016.

**Figure 3:**
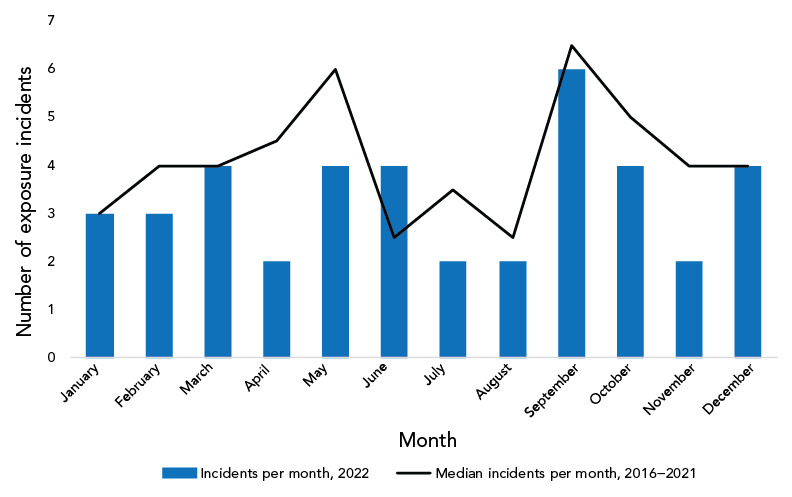
Seasonality analysis using median confirmed exposure incidents^a^ per month, Canada, 2016–2022 ^a^ Exposure incidents include those that involved a suspected or confirmed laboratory-acquired infection

**Figure 3** shows that in 2022, the number of confirmed exposure incidents was lowest in April, July, August and November (two incidents per month) and as was the case in previous years, the exposure incident rate was highest in September (six incidents per month).

### Exposure incidents by main activity and sector

In 2022, the most common activity being performed at the time of a reported exposure incident was microbiology (n=20; 50.0%), followed by *in vivo* animal research (n=9; 22.5%). Other less cited activities include animal care (n=3; 7.5%), cell culture (n=2; 5%), autopsy/necropsy (n=1; 2.5%), microscopy (n=1; 2.5%), other (n=3; 7.5%) and unknown (n=1; 2.5%). Definitions of activities can be found in the **Appendix**, **Table A1**.

**Figure 4:**
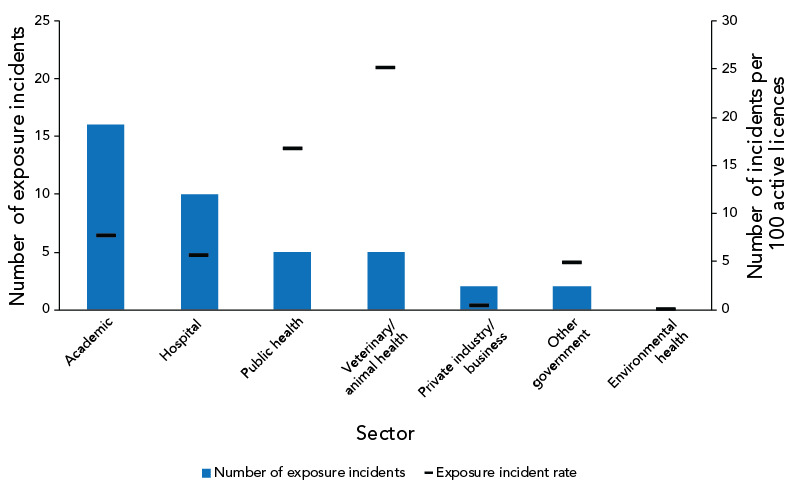
Confirmed exposure incidents and active licences by sector reported to Laboratory Incident Notification Canada, Canada, 2022

As shown in **Figure 4**, the majority of the reported confirmed exposure incidents in 2022 occurred in the academic sector (n=16; 40%), followed by the hospital sector (n=10; 25.0%). The sector with the highest number of exposure incidents per 100 active licences was the veterinary/animal health sector (25 exposure incidents per 100 active licences), followed by the public health sector (17 exposure incidents per 100 active licences).

### Implicated human pathogens and toxins

**Table 1** shows the distribution of biological agents (bacteria, fungus, parasite, prion, toxin, virus) involved in the exposure incidents reported in 2022 by risk group (RG2, RG3) and whether classified as SSBA. The majority of the 43 HPTs implicated in the 40 confirmed exposure reports were both non-SSBA (n=36; 83.7%) and human RG2 pathogens (n=27; 62.7%). Six SSBA agents were reported in 2022 (14.0%). Bacteria were the most reported agent type in 2022 (n=19; 44.2%), followed by fungus (n=10; 23.3%) and virus (n=7; 16.3%). One non-SSBA report involved parasites (2.3%). The most common RG2 agents involved in exposure incidents were *Neisseria meningitidis* (n=5; 11.6%) and Pertussis toxin (n=3; 7.0%). The most common RG3 agent involved was *Brucella melitensis* (n=3; 7.0%), followed by SARS-CoV-2 (n=2; 4.7%). *Escherichia coli* and *Coxiella burnetii* were the biological agents involved in the two suspected LAIs.

**Table 1:** Human pathogens or toxins involved in reported exposure incidents by risk group level and security sensitive status, Canada, 2022 (N=43)

Biological agent type by risk group	Non-SSBA		SSBA		Unknown		Total	
	n	%^a^	n	%	n	%	n	%
**RG2 agents**	**27**	**63**	**0**	**0**	**0**	**0**	**27**	**63**
Bacteria	15	35	0	0	0	0	15	35
Fungus	4	9	0	0	0	0	4	9
Parasite	1	2	0	0	0	0	1	2
Prion	1	2	0	0	0	0	1	2
Toxin	3	7	0	0	0	0	3	7
Virus	3	7	0	0	0	0	3	7
**RG3 agents**	**9**	**21**	**6**	**14**	**0**	**0**	**15**	**35**
Bacteria	0	0	4	9	0	0	4	9
Fungus	5	12	1	2	0	0	6	14
Parasite	0	0	0	0	0	0	0	0
Prion	1	2	0	0	0	0	1	2
Toxin	0	0	0	0	0	0	0	0
Virus	3	7	1	2	0	0	4	9
Unknown agents	0	0	0	0	1	2	1	2
**Total**	**36**	**84**	**6**	**14**	**1**	**2**	**43**	**100**

Abbreviations: RG, risk group; SSBA, security sensitive biological agent

^a^ Percentages are rounded to the nearest whole number

### Occurrence types

**Figure 5:**
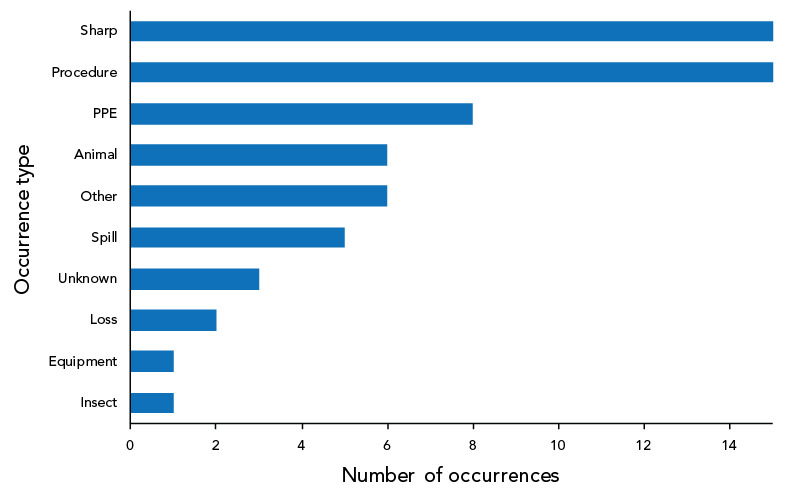
Reported occurrence types in confirmed exposure incidents, Canada, 2022 (N=62) Abbreviation: PPE, personal protective equipment

As shown in **Figure 5**, 62 occurrence types were cited in the 40 confirmed exposure incidents reported in 2022. Sharps and procedure-related incidents (n=15; 24.2% each) were the most reported type of occurrences, followed by personal protective equipment (PPE)-related incidents (n=8; 12.9%) and animal-related incidents (n=6; 9.7%). Definitions of occurrence types are provided in **Table A2**.

### Root causes

Through the investigation of follow-up reports, 84 root causes were identified (**Table 2**), resulting in an average of 2.1 root causes per confirmed exposure report. Human interaction was the most identified root cause (n=20; 23.8%), followed by issues with standard operating procedures (n=19; 22.6%). Training, communication and other root causes were the least common root causes reported (n=7; 8.3% each).

**Table 2:** Root causes reported in follow-up reports of confirmed exposure incidents, Canada, 2022 (N=84)

Root cause	Examples of areas of concern	Citations	
		n	%
Human interaction	A violation (cutting a corner, not following correct procedure, deviating from standard operating procedure)	20	23.8%
	An error (a mistake, lapse of concentration or slip of any kind)		
Standard operating procedure	Documents were followed as written but were not correct for activity/task	19	22.6%
	Procedures that should have been in place were not in place		
	Documents were not followed correctly		
Equipment	Equipment quality control needed improvement	14	16.7%
	Equipment failed		
	Equipment was not appropriate for purpose		
Training	Training not in place but should have been in place	7	8.3%
	Training not appropriate for task/activity		
	Staff were not qualified or proficient in performing task		
Communication	Communication did not occur but should have	7	8.3%
	Communication was unclear, ambiguous, etc.		
Management and oversight	Supervision needed improvement	10	11.9%
	Lack of auditing of standards, policies and procedures		
	Risk assessment needed improvement		
Other	Not applicable	7	8.3%

### Exposed individuals

A total of 93 individuals were exposed through the 40 exposure incidents reported and confirmed to LINC in 2022. Most exposed individuals held a bachelor’s degree (n=37; 39.8%), followed by a technical or a trade college diploma (n=27; 29.0%), a master’s degree (n=12; 12.9%) or were at high school level (n=7; 7.5%). Individuals with the highest education level, MD/PhD, were the least exposed to laboratory incidents (n=2; 2.2%).

**Figure 6:**
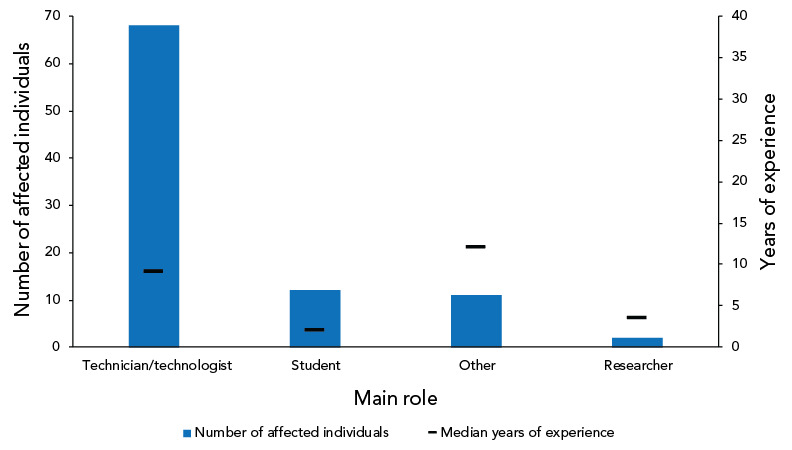
Individuals affected in exposure incidents reported by number of years of laboratory experience and main role^a^, Canada, 2022 (N=93) ^a^ Other roles are those which reporters feel are not captured in the other categories, such as clinical veterinarians

As shown in **Figure 6**, most exposed individuals worked as a technician/technologist (n=68; 73.1%), a student (n=12; 12.9%), on another role (n=11; 11.8%) or as a researcher (n=2; 2.2%). The median number of years of experience for technicians/technologists was nine while the median number of years of experience for students was two.

Most of the 93 exposed individuals were exposed to HPTs through inhalation (n=73; 78.5%) or inoculation/injection through needle/sharps (n=11; 11.8%) (data not shown). Other routes of exposure for the rest of exposed individuals include absorption via contact with mucous membrane, absorption via contact with skin and inoculation/injection through bite/scratch.

### Time between the incident and the reporting date

**Figure 7:**
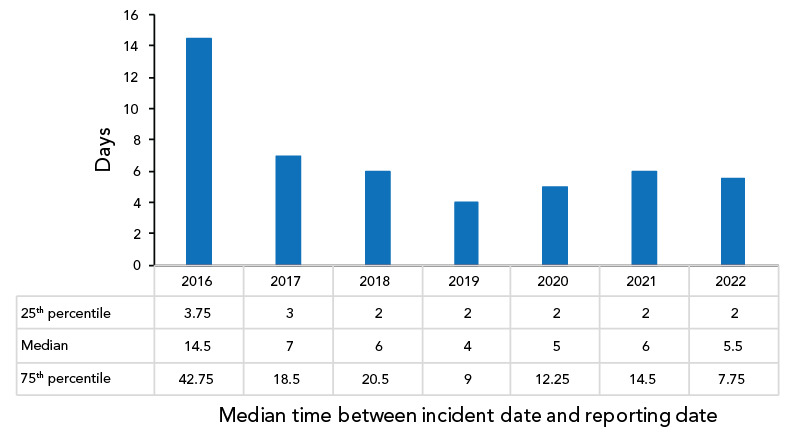
Time between the date of the incident and the date report was submitted to Laboratory Incident Notification Canada, Canada, 2016–2022

In 2022, 62.5% (n=25) of all confirmed exposure reports (n=40) were submitted to LINC within one week of the incident. The median number of days from incident occurrence to LINC reporting date was 5.5 days in 2022 (**Figure 7**), which is slightly shorter than the median delay of six days reported in 2020 and 2021.

## Discussion

Forty confirmed laboratory exposure incidents were reported to LINC in 2022. This marks a slight decrease from the 44 confirmed exposure incidents reported in 2021. Two of the exposure incidents in 2022 led to suspect LAIs. Similar to 2021, most of the exposure incidents in 2022 occurred while performing microbiology activities and in academic and hospital sectors. The exposures were most commonly due to sharps and procedural breaches. Most biological agents involved were RG2 and non-SSBAs, while bacteria were the most reported agent type.

The exposure incident rate was lower in 2022 (3.8 incidents per 100 active licences) compared to the previous year (4.3 incidents per 100 active licences). This decrease in the rate could be due to heightened vigilance in laboratories related to coronavirus disease 2019 (COVID-19) biosafety measures and better knowledge of laboratory safety practices.

### Increase in number of affected individuals

Ninety-three individuals were exposed to HPTs through 40 confirmed exposure incidents in 2022, which is an increase of 29% compared to 2021 (n=72). As in 2021, most of the affected individuals held the role of laboratory technicians/technologists. Technicians/technologists may also be the individuals who are most often in contact with HPTs in labs due to their qualifications or years of experience. While an exposure incident usually involves one to three individuals, further analysis of 2022 data showed that most of the individuals affected in 2022 were implicated through one specific laboratory incident, which affected 47 individuals by inhalation of *B. melitensis*, which is one of the most involved pathogens in laboratory-acquired infections ((11,12)).

### Decline in SARS-CoV-2 exposures

Last year marked the second full year of the COVID-19 pandemic. In contrast to 2021, when severe acute respiratory syndrome coronavirus 2 (SARS-CoV-2) was the most implicated agent across all pathogen groups due in part to heightened laboratory activities focused on COVID-19, in 2022, SARS-CoV-2 was the fourth most implicated agent across all pathogen groups. The reduction in the proportion of reported exposures involving SARS-CoV-2 agents compared to the previous years of the pandemic might be due to a return to more normal operations in laboratories, which includes the manipulation of other pathogens besides SARS-CoV-2 towards the end of 2022. With the expectation of potential new variants in the future, it is reasonable for the virus to continue to feature among the commonly reported agents ((13,14)). It is important to note that as per the HPTA, reported exposure incidents involving SARS-CoV-2 did not include exposure incidents related to diagnostic activities.

### Changes in seasonal exposure incidents trend

The median number of exposure incidents reported per month from 2016 to 2021 was lowest in June and August (2.5 incidents per month) and highest in September (6.5 incidents per month). The monthly occurrence of laboratory exposure incidents reported throughout 2022 followed a similar trend of the previous six years with a few exceptions. The number of exposure incidents reported remained highest in September 2022 (n=6); however, the number of exposure incidents was lowest in April, July, August and November (n=2 each). While the peak in September 2022 was expected and may be explained by the return of students and workers to laboratories after vacation in the summer months, the deviation from the normal trend observed for the low number of exposure incidents is notable. April and November 2022 had far fewer incidents reported than the median of the previous six years. The lower number of incidents in April 2022 may be explained by reduced laboratory staff due to sickness from the Omicron variant of the SARS-CoV-2 virus. It is possible that laboratory workers took more vacation in November following the easing of travel restrictions in October 2022.

### Human interaction remains the leading root cause of incidents

Human interaction, which includes violations and errors, remained the dominant root cause cited in 2022 and made up nearly 23.8% of the total number of root causes cited. The stress and fatigue experienced by workers in the context of the COVID-19 pandemic could be a contributing factor to incidents attributed to human interaction in laboratories ((15,16)); however, the proportion of human interaction citations decreased by 4% compared to 2021. This decrease could be due to improvements in laboratory practises as a result of the adoption of new COVID-19 measures and increased biosafety vigilance in laboratories. Issues with standard operating procedures, equipment, management, and oversight were also frequently reported as root causes of laboratory incidents.

## Strengths and limitations

The strength of the LINC program is that it allows for the collection of exposure incidents data from licensed laboratories across Canada through a standardized mandatory reporting system. The Public Health Agency of Canada’s Biosecurity Portal provides a user-friendly method to report laboratory exposure incidents and serves as a reliable source of data for analyzing exposure incident trends over time.

The possibility of under-reporting of laboratory exposure incidents remains a limitation that must be taken into consideration, as the rate of under-reporting is still unknown and can affect the results. To encourage the reporting of laboratory incidents, the Centre for Biosecurity offers an alternative method for incident reporting by email. Limited centralized information about laboratory incidents outside of Canada makes it challenging to compare trends in Canada with those of other countries. Within Canada, the COVID-19 pandemic has impacted normal laboratory operations and potentially affected the overall trend of laboratory exposure incidents across Canada. Data collected in the upcoming years will allow for a better understanding of trends and will clarify COVID-19’s impact on laboratory incidents in Canada.

Neither information about the number of laboratory employees nor their respective roles were collected during the reporting process. As such, the number of active licences was used as a proxy for workforce size. However, this limited analysis of the data and understanding of exposure incident rates. The location of laboratories involved in exposure reports also was not collected. Therefore, the information provided in this report should be used only at a national level. Finally, it should be noted that slight decreases or increases in the number of exposure incidents may be due to natural variability from one year to the next.

## Conclusion

Overall, the results observed in 2022 were similar in many respects to those from 2021, with a few exceptions. The exposure incident rate was lower in 2022 than in 2021; however, it remains unclear if this was a true decrease, as the full effect of the COVID-19 pandemic on laboratory operations can only be assessed after a couple of years. Sharp-related incidents and issues related to standard operating procedures remain the most common occurrence types, while human interaction remain the most cited root cause of exposure incidents.

## Appendix

**Table A1:** Definitions of main activity

Main activity	Description
Animal care	Activities such as attending to the daily care of animals and providing animals with treatment
Autopsy or necropsy	Post-mortem surgical examinations for purposes such as determining cause of death or to evaluate disease or injury for research or educational purposes
Cell culture	The process of growing cells under controlled conditions. It can also involve the removal of cells from an animal or plant
Education or training	Education or training of students and/or personnel on laboratory techniques and procedures
*In vivo* animal research	Experimentation with live, non-human animals
Maintenance	The upkeep, repair, and/or routine and general cleaning of equipment and facilities
Microbiology	Activities involving the manipulation, isolation, or analysis of microorganisms in their viable or infectious state
Molecular investigations	Activities involving the manipulation of genetic material from microorganisms or other infectious material for further analysis
Serology	Diagnostic examination and/or scientific study of immunological reactions and properties of blood serum
Hematology	Scientific study of the physiology of blood

**Table A2:** Definitions of occurrence type

Occurrence type	Description
Spill	Any unintended release of an agent from its container
Loss of containment	Includes malfunction or misuse of containment devices or equipment and other type of failures that results in the agent being spilled outside of, or released from, containment
Sharps-related	Needle stick, cut with scalpel, blade or other sharps injury (i.e. broken glass)
Animal-related	Includes animal bites or scratches, as well as other exposure incidents resulting from animal behavior (i.e. animal movement resulting in a needle stick)
Insect-related	Includes insect bites
PPE-related	Includes either inadequate PPE for the activity or failure of the PPE in some way
Equipment-related	Includes failure of equipment, incorrect equipment for the activity, or misuse of equipment
Procedure-related	Includes instances when written procedures were not followed, were inadequate or absent, or were incorrect for the activity

Abbreviation: PPE, personal protective equipment
